# Supplementary material for: The enteric pathogen Cryptosporidium parvum exports proteins into the cytosol of the infected host cell
Source: eLife. 2021 Dec 6;10:e70451. doi: 10.7554/eLife.70451 (PMC8687662; doi:10.7554/eLife.70451)
Supplement: Source code 1. [file elife-70451-supp2.zip › Supplemental Code File.pdf]

Supplemental code file for mansucript titled: The diarrheal pathogen Cryptosporidium parvum exports proteins into the cytoplasm of the infected host cell

Jennifer E. Dumaine, Adam Sateriale, Alexis R. Gibson, Amita Reddy, Jodi Gullicksrud, Emma Hunter, Joseph Clark, Boris Striepen

produced on 06-04-2021

Contents

1 R packages used in this analysis 1

2 Processing raw reads (kallisto) 2

3 Using R to import and analyze data 2

4 Identification of differential expression in Medle2 expressing cells 3

5 Enrichment analysis 5

6 Examining the Medle2 signature in other datasets 5

7 Session Info 8

1 R packages used in this analysis

All packages used are available from CRAN, Bioconductor or GitHub. Raw data used in this analysis can be accessed from the Gene Expression Omnibus (GEO) accession number GSE174117.

```
library(dplyr)
library(biomaRt)
library(readr)
library(Biostrings)
library(tximport) #package for getting Kallisto results into R
library(ensembldb)
library(EnsDb.Hsapiens.v86)
library(ggplot2)
library(gplots)
library(reshape2)
library(genefilter)
library(RColorBrewer)
library(limma)
library(edgeR)
library(Biobase)
library(tidyverse)
```

2 Processing raw reads (kallisto)

Raw reads were pseudoaligned with kallisto the following code was put directly into Terminal. myHumanindex was built from EnsemblDB version 86 of the human genome.

```
kallisto quant -i myHumanindex -o GFP_untrans_rep1 -t 4 -b 60 --single -l 250 -s 30 GFP_untrans_1_merge

kallisto quant -i myHumanindex -o GFP_untrans_rep2 -t 4 -b 60 --single -l 250 -s 30 GFP_untrans_2_merge

kallisto quant -i myHumanindex -o GFP_untrans_rep3 -t 4 -b 60 --single -l 250 -s 30 GFP_untrans_3_merge

kallisto quant -i myHumanindex -o M2_untrans_rep1 -t 4 -b 60 --single -l 250 -s 30 M2_untrans_1_merged.

kallisto quant -i myHumanindex -o M2_untrans_rep2 -t 4 -b 60 --single -l 250 -s 30 M2_untrans_2_merged.

kallisto quant -i myHumanindex -o M2_untrans_rep3 -t 4 -b 60 --single -l 250 -s 30 M2_untrans_3_merged.

kallisto quant -i myHumanindex -o GFP_rep1 -t 4 -b 60 --single -l 250 -s 30 GFP_1_merged.fastq.gz

kallisto quant -i myHumanindex -o GFP_rep2 -t 4 -b 60 --single -l 250 -s 30 GFP_2_merged.fastq.gz

kallisto quant -i myHumanindex -o GFP_rep3 -t 4 -b 60 --single -l 250 -s 30 GFP_3_merged.fastq.gz

kallisto quant -i myHumanindex -o M2_rep1 -t 4 -b 60 --single -l 250 -s 30 M2_1_merged.fastq.gz

kallisto quant -i myHumanindex -o M2_rep2 -t 4 -b 60 --single -l 250 -s 30 M2_2_merged.fastq.gz

kallisto quant -i myHumanindex -o M2_rep3 -t 4 -b 60 --single -l 250 -s 30 M2_3_merged.fastq.gz
```

3 Using R to import and analyze data

```r
listTables(EnsDb.Hsapiens.v86)
# now take a look at the columns of the "tx" table within the database
listColumns(EnsDb.Hsapiens.v86, "tx")

#use the 'transcripts' function from the EnsemblDB package to get annotation info
Tx <- transcripts(EnsDb.Hsapiens.v86,
                  columns=c(listColumns(EnsDb.Hsapiens.v86,
                                        "tx"), "gene_name"))

Tx <- as.data.frame(Tx)
head(Tx)
dim(Tx)

#need to change first column name to 'target_id'
Tx <- dplyr::rename(Tx, target_id = tx_id)
row.names(Tx) <- NULL
head(Tx)
Tx <- Tx[,c(6,12)] #transcript id is now the first column

targets <- read.table("jennieStudydesign", row.names=NULL, header = T, as.is = T)
files <- file.path(targets$sample, "abundance.h5")
```

```
all(file.exists(files))

Txi_gene_all <- tximport(files,
                     type = "kallisto",
                     tx2gene = Tx,
                     txOut = FALSE,
                     countsFromAbundance = "lengthScaledTPM",
                     ignoreTxVersion = TRUE)
```

Study design file

4 Identification of differential expression in Medle2 expressing cells

```
groups <- targets$treatment
groups <- factor(groups)
sampleLabels <- targets$sample
colnames(Txi_gene_all$abundance) <-sampleLabels
DGEList <- DGEList(Txi_gene_all$counts)
cpm <- cpm(DGEList)
log2.cpm <- cpm(DGEList, log=TRUE)
table(rowSums(DGEList$counts==0)==12)
#data was filtered
keepers <- rowSums(cpm>1)>=3 #removed all genes with expression less than 1 in at least 3 samples
DGEList.filtered <- DGEList[keepers,]
#after filtering data was normalized
DGEList.filtered.norm <- calcNormFactors(DGEList.filtered, method = "TMM")
log2.cpm.filtered.norm <- cpm(DGEList.filtered.norm, log=TRUE)
write.table(log2.cpm.filtered.norm, "norm_filtered.txt", quote= FALSE, sep = "\t")
#normalized data was input for GSEA analysis
#set up your design matrix
groups
groups <- relevel(groups, "GFP_trans") #may need to use 'relevel' function
design <- model.matrix(~0 + groups)
#normalize your data using the mean-variance relationship using the VOOM function from Limma
v.DGEList.filtered.norm <- voom(DGEList.filtered.norm, design, plot = TRUE)
# fit a linear model to your data
fit <- lmFit(v.DGEList.filtered.norm, design)

# Contrast matrix
#how do cells respond to transfection wih Medle2?
contrast.matrix <- makeContrasts(Medle2 = groupsM2_trans - groupsGFP_trans,
                                 levels=design)
# extract the linear model fit -----
fits <- contrasts.fit(fit, contrast.matrix)
#get bayesian stats for your linear model fit
ebFit <- eBayes(fits)
# TopTable to view DEGs -----
myTopHits <- topTable(ebFit, adjust ="BH", coef=1, number= 5000, sort.by="logFC", p.value=0.05)
myTopHits <- as_tibble(myTopHits, rownames = "geneSymbol")
ER.Stress.LE <- subset(myTopHits,
```

```
                   geneSymbol=="NUPR1" |
                   geneSymbol=="CREB3L1" |
                   geneSymbol== "FBXO2" |
                   geneSymbol== "BBC3" |
                   geneSymbol== "RASGRF2" |
                   geneSymbol== "SERP2" |
                   geneSymbol== "DDIT3" |
                   geneSymbol== "GET4" |
                   geneSymbol== "CHAC1" |
                   geneSymbol== "PIGBOS1" |
                   geneSymbol== "TRIB3" |
                   geneSymbol== "EXTL1" |
                   geneSymbol== "ATP2A1" |
                   geneSymbol== "MAP3K5" |
                   geneSymbol== "TMUB1" |
                   geneSymbol== "DNAJB2" |
                   geneSymbol== "USP13" |
                   geneSymbol== "DNAJC18" |
                   geneSymbol== "GRINA" |
                   geneSymbol== "LRRK2")
```

```
ggplot(myTopHits, aes(x=logFC, y=-log10(adj.P.Val))) + geom_point(size=2) +
  geom_point(shape = 1,size = 2,colour = "black") +
  geom_point(mapping =NULL, ER.Stress.LE, size = 2, colour= "#FF2600", inherit.aes = TRUE) +
  theme_linedraw() +
  geom_text(inherit.aes = TRUE, data=ER.Stress.LE, label=ER.Stress.LE$geneSymbol) +
  theme(axis.title = element_blank(),
        panel.grid.major = element_blank(),
        panel.grid.minor = element_blank(),
        legend.text = element_text(size = 15),
        axis.text.x = element_text(size=14, colour = "black"),
        axis.text.y = element_text(size=14, colour = "black"),
        plot.title = element_text(size=20, colour = "black")) +
  ylim(-0.5,8) +
  xlim(-8,8) +
  geom_hline(yintercept = -log10(0.05), linetype="dashed", colour="grey", size=1) +
  geom_vline(xintercept = 0, linetype="dashed", colour="grey", size=1)
```

```
head(v.DGEList.filtered.norm$E)
results <- decideTests(ebFit, method="global", adjust.method="BH", p.value=0.01, lfc=1)
colnames(v.DGEList.filtered.norm$E) <- sampleLabels
diffGenes <- v.DGEList.filtered.norm$E[results[,1] !=0,]

# Heatmap
myheatcol <- colorRampPalette(colors=c("#0095FF","white","#FF2600"))(100)
hr <- hclust(as.dist(1-cor(t(diffGenes), method="pearson")), method="complete")
#cluster rows by pearson correlation
hc <- hclust(as.dist(1-cor(diffGenes, method="spearman")), method="complete")
#cluster columns by spearman correlation
mycl <- cutree(hr, k=2)
mycolhc <- rainbow(length(unique(mycl)), start=0.1, end=0.9)
mycolhc <- mycolhc[as.vector(mycl)]
```

```
#plot the hclust results as a heatmap
heatmap.2(diffGenes, Rowv=as.dendrogram(hr), Colv=NA,
          col=myheatcol, scale='row', labRow=NA,
          density.info="none", trace="none", RowSideColors=mycolhc,
          cexRow=1, cexCol=1, margins=c(8,20))
```

5 Enrichment analysis

For enrichment analysis the normalized expression file was used to generate the .gct file for input into GSEA software. Gene sets from the C5: Gene Ontology database of MSigDB signatures were used.

6 Examining the Medle2 signature in other datasets

The Medle2 signature was generated from the list of differentially expressed genes wih a p-value < 0.01.

```
# The following code was used to format data from previously published RNAseq studies.
library(SeqGSEA)
library(tidyverse)
library(dplyr)
library(Seurat)
library(dplyr)
library(Matrix)

## For scRNAseq from Miniguts
path.data <- '/Users/katelynwalzer/Desktop/Single_cell_transcriptomics/Miniguts/raw_counts.tsv'
#raw_counts.tsv.gz

#Way to read in data for Seurat
#Replace "," with "\t" for tsv
#raw_counts<-read.table(file=paste0("/Users/nd48/Desktop/seurat/realData/","all_merged.csv"),sep=",")

#head(raw_counts)

#mydata <- CreateSeuratObject(raw.data = raw_counts, min.cells = 3, min.genes = 200,
#project = "mydata_scRNAseq")

#mydata

##### Load raw data #####
raw.data <- as.matrix(read.csv(file = path.data, header = T, sep = "\t"))

##### QC, normalize #####
SO.tmp <- CreateSeuratObject(counts = raw.data, min.cells = 4, min.genes = 300,
                             project = "OrganoidsAndMiniguts")

SO.tmp[["percent.mt"]] <- PercentageFeatureSet(SO.tmp, pattern = "^mt-")

#Set filtering criteria for seurat objects
#Filter cells with mitochondria below 4 percent and above 15 percent
#Filter cells less than 2500 and above 5000 number of genes
min.features <- 2500
```

```
max.features <- 5000


SO.tmp <- subset(SO.tmp, nFeature_RNA > min.features & nFeature_RNA <
                   max.features & percent.mt < 15 & percent.mt > 4)
#Checked, these numbers match the paper

SO.tmp <- NormalizeData(object = SO.tmp, normalization.method = "LogNormalize",
                        scale.factor = 10000)

list.of.seurats <- SplitObject(SO.tmp, split.by = "ident")

for (i in 1:length(list.of.seurats)) {
  list.of.seurats[[i]] <- FindVariableFeatures(list.of.seurats[[i]],
                                               selection.method = "vst",
                                               nfeatures = 2000, verbose = FALSE)
}

VariableFeaturePlot(list.of.seurats[[i]]) #Plot variable features without labels

seurat.anchors <- FindIntegrationAnchors(object.list = list.of.seurats, dims = 1:30)

seurat.integrated <- IntegrateData(anchorset = seurat.anchors, dims = 1:30)

#Switch to integrated assay, not RNA
DefaultAssay(seurat.integrated) <- "integrated"

#Run standard workflow
seurat.integrated <- ScaleData(seurat.integrated, verbose = FALSE)


#Really only need up to here for expression matrix, I just wanted to see everything else!

seurat.integrated <- RunPCA(seurat.integrated, verbose = FALSE, npcs = 40)
DimPlot(seurat.integrated, reduction = "pca", group.by = "orig.ident")

seurat.integrated <- FindNeighbors(seurat.integrated, k.param = 10, dims = 1:11)
seurat.integrated <- FindClusters(seurat.integrated, resolution = 0.7)
head(Idents(seurat.integrated), 5)

seurat.integrated <- RunUMAP(seurat.integrated, dims = 1:11)
DimPlot(seurat.integrated, reduction = "umap")
DimPlot(seurat.integrated, reduction = "umap", group.by = "orig.ident")



#Making expression matrix
library(data.table)
write_this_out_exp_file <- as.data.frame(as.matrix
                                         (seurat.integrated@assays[["integrated"]]@scale.data))
fwrite(x = write_this_out_exp_file, row.names = TRUE,
       file = "2021_3_6_expression_matrix_integrated_data_all.csv")
```

```r
#Do each sample individually
MinigutCrypto <- subset(seurat.integrated, subset = orig.ident == "MinigutCrypto")
MinigutYoung <- subset(seurat.integrated, subset = orig.ident == "MinigutYoung")

#Now want individual expression matrix
write_this_out_MinigutCrypto_only <- as.data.frame(as.matrix
                                                  (MinigutCrypto@assays[["integrated"]]@scale.data))
fwrite(x = write_this_out_MinigutCrypto_only, row.names = TRUE,
       file = "2021_3_6_expression_matrix_integrated_data_MinigutCrypto.csv")

write_this_out_MinigutYoung_only <- as.data.frame(as.matrix
                                                 (MinigutYoung@assays[["integrated"]]@scale.data))
fwrite(x = write_this_out_MinigutYoung_only, row.names = TRUE,
       file = "2021_3_6_expression_matrix_integrated_data_MinigutYoung.csv")

MinigutCrypto <- read.csv
("Users/Alexis/Desktop/classProject/2021_3_6_expression_matrix_integrated_data_MinigutCrypto.csv")

##make a collapsed object,enterocyte only----
MinigutCrypto_select <- select(MinigutCrypto, matches("Gene Symbol"), contains("Enterocytes"))
write.table(MinigutCrypto_select, "MinigutCrypto.txt" ,row.names = FALSE, col.names = TRUE, sep = "\t")
MinigutYoung_select <- select(MinigutYoung, matches("Gene Symbol"), contains("Enterocytes"))
write.table(MinigutYoung_select, "MinigutYoung.txt" ,row.names = FALSE, col.names = TRUE, sep = "\t")

# For RNAseq of rotavirus infection
rotavirus <- read.table("rotavirus_2017.txt", header=TRUE)
row.names(rotavirus) <- NULL
Symbols <- convertEnsembl2Symbol(rotavirus$Geneid)
rotavirus<- as_tibble(rotavirus)

listTables(EnsDb.Hsapiens.v86)
listColumns(EnsDb.Hsapiens.v86, "gene")
GI <- genes(EnsDb.Hsapiens.v86,
                 columns=c(listColumns(EnsDb.Hsapiens.v86,
                                       "gene_id"), "gene_name"))
GI <- as.data.frame(GI)
row.names(GI) <- NULL
head(GI)
GI <- GI[,c(6,7)]
write.table(GI, "86_id_symbol.txt", col.names = TRUE)
names(rotavirus)[names(rotavirus) == "Geneid"] <- "gene_id"


rota <- left_join(rotavirus, GI)
write.table(rota, "rotavirus_17_Symbol", col.names = TRUE)

# For RNAseq of Caco-2 infected with Salmonella
Caco2_mock_1 <- read.table("GSM4056572_Caco2_mock_1.txt", header=TRUE)
Caco2_GFPpos_1 <- read.table("GSM4056573_Caco2_GFP+_1.txt", header=TRUE)
Caco2_GFPneg_1 <- read.table("GSM4056574_Caco2_GFP-_1.txt", header=TRUE)

Caco2_mock_2 <- read.table("GSM4056582_Caco2_mock_2.txt", header=TRUE)
Caco2_GFPpos_2 <- read.table("GSM4056583_Caco2_GFP+_2.txt", header=TRUE)
```

```
Caco2_GFPneg_2 <- read.table("GSM4056584_Caco2_GFP-_2.txt", header=TRUE)

Caco2_GFPneg_1 <- dplyr::rename(Caco2_GFPneg_1, RPKM_GFP01 = RPKM_GFP.1)
Caco2_GFPneg_2 <- dplyr::rename(Caco2_GFPneg_2, RPKM_GFP02 = RPKM_GFP.2)

Caco1_mock_GFPpos_1 <- left_join(Caco2_mock_1, Caco2_GFPpos_1)
Caco2_1 <- left_join(Caco2_mock_GFPpos_1, Caco2_GFPneg_1)

Caco2_mock_GFPpos_2 <- left_join(Caco2_mock_2, Caco2_GFPpos_2)
Caco2_2 <- left_join(Caco2_mock_GFPpos_2, Caco2_GFPneg_2)

Caco2_1 <- dplyr::rename(Caco2_1, mock1 = RPKM_mock1, GFPpos_1 = RPKM_GFP.1,
                         GFPneg_01 = RPKM_GFP01)
Caco2_2 <- dplyr::rename(Caco2_2, mock2 = RPKM_mock2, GFPpos_2 = RPKM_GFP.2,
                         GFPneg_2 = RPKM_GFP02)

Hs.trans <- readDNAStringSet("Homo_sapiens.GRCh38.cdna.all.fa")
slotNames(Hs.trans) #look at the 'slots' in the object you just created
Hs.trans@ranges@NAMES #access one of these slots
#replace transcript names with new names that don't have decimal version number
names(Hs.trans) <- gsub("(\\.).*", "", names(Hs.trans))
writeXStringSet(Hs.trans, "Hs.trans.shortnames.fasta")

Caco2_ID <- gsub("(\\.).*", "", Caco2_1$GeneID)
class(Caco2_2)
Caco2_ID <- as.data.frame(Caco2_ID)
Caco2_ID <- dplyr::rename(Caco2_ID, GeneID = GeneIDver)

Caco2_IDS <- left_join(Caco2_ID, Caco2_1, by.x="GeneIDver",by.y="GeneID")
write.table(Caco2_ID, "Caco2_nover", col.names = TRUE)
Caco2_toMerge <- read.table("Caco2_no_ver.txt", header=TRUE)
Caco2_toMerge <- dplyr::rename(Caco2_toMerge, GeneID = GeneIDver,GeneIDver = GeneID)
Caco2_IDS <- left_join(Caco2_1, Caco2_toMerge)
Caco2_all <- left_join(Caco2_IDS, Caco2_2)
write.table(Caco2_all, "Caco2_Salmonella", col.names = TRUE, row.names = FALSE)
Caco2_Sal <- read.table("Caco2_Sal.txt", header=TRUE)
GI <- dplyr::rename(GI, GeneID = gene_id)
Caco2 <- left_join(Caco2_Sal, GI)
write.table(Caco2, "Caco2_Salmonella", col.names = TRUE, row.names = FALSE)
```

The data formatted above was then input into the GSEA software and the Medle2 signature was input as the gene set to be evaluated.

7 Session Info

```
R version 3.6.2 (2019-12-12)
Platform: x86_64-apple-darwin15.6.0 (64-bit)
Running under: macOS  10.16

Matrix products: default
LAPACK: /Library/Frameworks/R.framework/Versions/3.6/Resources/lib/libRlapack.dylib
```

```
locale:
[1] en_US.UTF-8/en_US.UTF-8/en_US.UTF-8/C/en_US.UTF-8/en_US.UTF-8

attached base packages:
[1] stats4    parallel  stats     graphics  grDevices utils     datasets  methods
[9] base

other attached packages:
 [1] genefilter_1.68.0        reshape2_1.4.4           EnsDb.Hsapiens.v86_2.99.0
 [4] ensembldb_2.10.2         AnnotationFilter_1.10.0  GenomicFeatures_1.38.2
 [7] AnnotationDbi_1.48.0     Biobase_2.46.0           GenomicRanges_1.38.0
[10] GenomeInfoDb_1.22.1      tximport_1.14.2          Biostrings_2.54.0
[13] XVector_0.26.0           IRanges_2.20.2           S4Vectors_0.24.4
[16] BiocGenerics_0.32.0      readr_1.4.0              biomaRt_2.42.1
[19] dplyr_1.0.2              RColorBrewer_1.1-2       gplots_3.1.0
[22] ggplot2_3.3.2            edgeR_3.28.1             limma_3.42.2

loaded via a namespace (and not attached):
 [1] ProtGenerics_1.18.0         matrixStats_0.57.0          bitops_1.0-6
 [4] bit64_4.0.5                 progress_1.2.2              httr_1.4.2
 [7] tools_3.6.2                 backports_1.2.0             R6_2.5.0
[10] KernSmooth_2.23-18          lazyeval_0.2.2              DBI_1.1.0
[13] colorspace_1.4-1            withr_2.3.0                 tidyselect_1.1.0
[16] prettyunits_1.1.1           bit_4.0.4                   curl_4.3
[19] compiler_3.6.2              DelayedArray_0.12.3         rtracklayer_1.46.0
[22] labeling_0.4.2              caTools_1.18.0              scales_1.1.1
[25] askpass_1.1                 rappdirs_0.3.1              stringr_1.4.0
[28] digest_0.6.27               Rsamtools_2.2.3             rmarkdown_2.5
[31] pkgconfig_2.0.3             htmltools_0.5.0             dbplyr_2.0.0
[34] rlang_0.4.8                 rstudioapi_0.12             RSQLite_2.2.1
[37] farver_2.0.3                generics_0.1.0              BiocParallel_1.20.1
[40] gtools_3.8.2                RCurl_1.98-1.2              magrittr_1.5
[43] GenomeInfoDbData_1.2.2      Matrix_1.2-18               Rcpp_1.0.5
[46] munsell_0.5.0               lifecycle_0.2.0             stringi_1.5.3
[49] yaml_2.2.1                  SummarizedExperiment_1.16.1 zlibbioc_1.32.0
[52] plyr_1.8.6                  BiocFileCache_1.10.2        grid_3.6.2
[55] blob_1.2.1                  crayon_1.3.4                lattice_0.20-41
[58] splines_3.6.2               annotate_1.64.0             hms_0.5.3
[61] locfit_1.5-9.4              knitr_1.30                  pillar_1.4.6
[64] ggsignif_0.6.0              XML_3.99-0.3                glue_1.4.2
[67] packrat_0.5.0               evaluate_0.14.1             vctrs_0.3.4
[70] gtable_0.3.0                openssl_1.4.3               purrr_0.3.4
[73] tidyr_1.1.2                 assertthat_0.2.1            xfun_0.23
[76] xtable_1.8-6                broom_0.7.2                 survival_3.2-7
[79] tibble_3.0.4                GenomicAlignments_1.22.1    memoise_1.1.0
[82] ellipsis_0.3.1
```
