# Supplementary material for: The enteric pathogen Cryptosporidium parvum exports proteins into the cytosol of the infected host cell
Source: eLife. 2021 Dec 6;10:e70451. doi: 10.7554/eLife.70451 (PMC8687662; doi:10.7554/eLife.70451)
Supplement: Transparent reporting form [file elife-70451-transrepform1.docx]

***eLife’s* transparent reporting form**

We encourage authors to provide detailed information *within their submission* to facilitate the interpretation and replication of experiments. Authors can upload supporting documentation to indicate the use of appropriate reporting guidelines for health-related research (see [EQUATOR Network](http://www.equator-network.org/%20)), life science research (see the [BioSharing Information Resource](https://biosharing.org/)), or the [ARRIVE guidelines](http://www.plosbiology.org/article/info:doi/10.1371/journal.pbio.1000412) for reporting work involving animal research. Where applicable, authors should refer to any relevant reporting standards documents in this form.

If you have any questions, please consult our Journal Policies and/or contact us: [editorial@elifesciences.org](mailto:editorial@elifesciences.org).

**Sample-size estimation**

- You should state whether an appropriate sample size was computed when the study was being designed
- You should state the statistical method of sample size computation and any required assumptions
- If no explicit power analysis was used, you should describe how you decided what sample (replicate) size (number) to use

Please outline where this information can be found within the submission (e.g., sections or figure legends), or explain why this information doesn’t apply to your submission:

We did not use power analysis to determine sample size for imaging quantification experiments, but during study design established a minimum of 14 images would be captured for each blinded condition using a scanning function to avoid capture bias. The number of cells quantified in the 14 images captured for these analyses is stated in the text of the results section and figure legends for Figures 2B, 2C, 3E. We decided to use one cage of 3 mice each for qPCR experiments on intestinal sections from infected and uninfected mice (Figure 6F), as this is what our breeding colony had available as age and gender matched controls. This is reported in the Methods section.

**Replicates**

- You should report how often each experiment was performed
- You should include a definition of biological versus technical replication
- The data obtained should be provided and sufficient information should be provided to indicate the number of independent biological and/or technical replicates
- If you encountered any outliers, you should describe how these were handled
- Criteria for exclusion/inclusion of data should be clearly stated
- High-throughput sequence data should be uploaded before submission, with a private link for reviewers provided (these are available from both GEO and ArrayExpress)

Please outline where this information can be found within the submission (e.g., sections or figure legends), or explain why this information doesn’t apply to your submission:

We define technical replicates as repeated measures of the same sample compared with biological replicates that are measures of separately generated samples. All slides prepared for microscopy analysis were prepared in duplicate technical replicates and experiments were repeated for a minimum of two biological replicates. This information can be found in the Methods section under the “In vitro infection and Immunofluorescence Assay” and “Immunohistochemistry on infected intestine” sections. Live imaging experiments were repeated as 3 biological replicates, and this is detailed in the Methods section. Western Blots were performed as 2 technical replicates on 2 biological samples. Flow cytometry results and the RNA sequencing dataset were generated from 3 biological replicates of transfected cells and the resulting dataset is available under GEO accession number GSE174117 (<https://www.ncbi.nlm.nih.gov/geo/query/acc.cgi?acc=GSE174117>)

as described in the Methods section. All qPCR experiments were carried out with 3 technical replicates per experiment. This is found in the Methods section.

**Statistical reporting**

- Statistical analysis methods should be described and justified
- Raw data should be presented in figures whenever informative to do so (typically when N per group is less than 10)
- For each experiment, you should identify the statistical tests used, exact values of N, definitions of center, methods of multiple test correction, and dispersion and precision measures (e.g., mean, median, SD, SEM, confidence intervals; and, for the major substantive results, a measure of effect size (e.g., Pearson's r, Cohen's d)
- Report exact p-values wherever possible alongside the summary statistics and 95% confidence intervals. These should be reported for all key questions and not only when the p-value is less than 0.05.

Please outline where this information can be found within the submission (e.g., sections or figure legends), or explain why this information doesn’t apply to your submission:

The information for statistical reporting, including statistical tests used, n values, and p values can be found in the text of the results section, as well as in the figure legends for Figures 2B and 3E. The p values used to determine a threshold for significance for the GSEA analysis were described in the text of the results section and in the figure legends for Figures 6C, 6D, and 6E.

(For large datasets, or papers with a very large number of statistical tests, you may upload a single table file with tests, Ns, etc., with reference to sections in the manuscript.)

**Group allocation**

- Indicate how samples were allocated into experimental groups (in the case of clinical studies, please specify allocation to treatment method); if randomization was used, please also state if restricted randomization was applied
- Indicate if masking was used during group allocation, data collection and/or data analysis

Please outline where this information can be found within the submission (e.g., sections or figure legends), or explain why this information doesn’t apply to your submission:

Images for the Brefeldin A quantification experiments Figure 3E were captured from masked slides using a scanning function to avoid bias during acquisition. Macro analysis using the ImageJ software was used to quantify captured images and then the group allocation (BFA treated or untreated) was unblinded for Figure 3E. This information is described in the Methods under the Quantification and Statistical Methods section. Infected and uninfected groups were allocated for Figure 6F as individual cages of 3 mice. This is described in the figure legend for Figure 6F.

**Additional data files (“source data”)**

- We encourage you to upload relevant additional data files, such as numerical data that are represented as a graph in a figure, or as a summary table
- Where provided, these should be in the most useful format, and they can be uploaded as “Source data” files linked to a main figure or table
- Include model definition files including the full list of parameters used
- Include code used for data analysis (e.g., R, MatLab)
- Avoid stating that data files are “available upon request”

Please indicate the figures or tables for which source data files have been provided:

The numerical source data has for the microscopy quantification experiments displayed in Figures 2B, 2C, and 3E has been provided as excel files. The code used for the RNAseq analysis displayed in Figure 6 is provided in the source data. Additionally, the MEDLE2 transfection dataset generated for Figure 6 was deposited under GEO accession number GSE174117. We have also provided excel sheets with summaries of the GSEA results for the MEDLE2 transfection dataset displayed in Figure 6D and the *Cryptosporidium* infection dataset displayed in Figure 6E.
